# Response from Varani et al. to “Comment on ‘the IS*6* family, a clinically important group of insertion sequences including IS26’ by Ruth M. Hall”

**DOI:** 10.1186/s13100-021-00258-8

**Published:** 2022-01-03

**Authors:** Alessandro Varani, Susu He, Patricia Siguier, Karen Ross, Michael Chandler

**Affiliations:** 1 School of Agricultural and Veterinary Sciences, Universidade Estadual Paulista, Jaboticabal, Sao Paulo Brazil; 2 State Key Laboratory of Pharmaceutical Biotechnology, Medical School of Nanjing University, Nanjing, 210093 Jiangsu China; 3 Centre de Biologie Intégrative-Université Paul SABATIER, CNRS - Laboratoire de Microbiologie et Génétique Moléculaires, UMR 5100 - bât. CNRS-IBCG, Toulouse, France; 4 Department of Biochemistry, Molecular and Cellular Biology, Protein Information Resource, Georgetown University Medical Center, Washington, DC USA; 5 Department of Biochemistry, Molecular and Cellular Biology, Georgetown University Medical Center, Washington, DC USA

## Abstract

The IS*6* family of insertion sequences is a large but coherent group which was originally named to avoid confusion between a number of identical or nearly identical IS that were identified at about the same time and given different names (IS*15*D, IS*26*, IS*46*, IS*140*, IS*160*, IS*176*). The underlying common mechanistic feature of all IS*6* family members which have been investigated is that they appear to transpose by replicative transposition and form pseudo compound transposons with the flanking IS in direct repeat and in which associated genes are simply transferred to the target replicon and lost from the donor.

In the accompanying letter Hall raises a number of very serious and wide-ranging criticisms of our recent review article concerning the IS*6* family of insertion sequences. She clearly feels that we have undervalued her work and that we question or ignore certain of her in vivo results. This impression is almost certainly the result of the standard of proof we generally apply to mechanistic aspects of transposition where we think it important to identify transposition intermediates including the types of synaptic, strand cleavage and strand transfer complexes involved.

## Background

Hall raises a number of serious and wide-ranging criticisms of our review article [1] (see also https://tnpedia.fcav.unesp.br/index.php/IS_Families/IS6_family) that we: 1) “seriously misrepresented” her work with Harmer; 2) failed to take into account her data [2]; 3) “incorrectly implied that any mechanism established for IS*26* can be assumed to apply to a range of IS that are at best very distantly related” and 4) that “questioned the existence of translocatable unit (TU)”. It was not our intention to question her results. We apologise if she has interpreted our text in this manner and are grateful for her exquisitely detailed and rigorous inspection of our review.

## Main text

1. It was certainly not our intention to wilfully misrepresent Hall’s data – which include excellent and incisive experiments and analyses. The impression that she has, merely reflects the fact that we have been consistently prudent and cautious in our interpretation of in vivo transposition data until such time as the intermediates in the process, in this case of conservative integration, have been identified. This can be seen in other IS family chapters of TnPedia (https://tnpedia.fcav.unesp.br/index.php/IS_Families [3];). It is especially important in cases where new mechanisms are proposed. Our standard of proof would entail identification of the types of synaptic, strand cleavage and strand transfer complexes involved, which could be accessed either from in vivo or in vitro studies but at present these are not available.
2. We acknowledge that one reference from the large body of Hall’s IS*26* work is missing: [2] (reference (6) in Hall’s letter) for which one of us, MC, had already apologised in personal correspondence. Reference (2), indeed, presents evidence that targeted integration does not *require* both IS ends, raising the possibility that the potential intermediate (Fig. 11 iii), (https://tnpedia.fcav.unesp.br/index.php/File:FigIS6.14.png) could be resolved in a number of different ways (replication, Holiday junction resolution etc.) as we have clearly stated (page 13,right column, lines 40–41) “The authors propose a model in which a single IS end is cleaved and transferred to the flank of the target IS end, an event which creates a Holliday junction, which, on branch migration, is resolved”. This further underlines the importance of directly identifying intermediate structures in confirming the proposed mechanism.
3. Concerning the IS family definition and its relation to mechanism: In developing ISfinder (https://www-is.biotoul.fr/index.php) and, more recently, TnPedia (https://tnpedia.fcav.unesp.br/index.php/Main_Page) and TnCentral [3], we have always adhered to the principle that, in addition to sequence similarities, members of a given family generally share a common basic transposition mechanism (defined in [4] and https://tnpedia.fcav.unesp.br/index.php/General_Information/IS_Identification). In the case of all IS*6* family members investigated thus far [1], that common feature appears to be replicative cointegrate formation and the formation of pseudo compound transposons [5] with the flanking IS in direct repeat in which associated genes are simply transferred to the target replicon and lost from the donor [5]. We did not mean to imply that the entire IS*6* family also possess the mechanistic variation proposed by Hall (i.e., targeted conservative integration). That has clearly yet to be addressed. Consequently, to continue to provide a consistent structural framework for understanding the relationship between the nearly 6000 IS in the ISfinder database, we continue to adhere to these rules and include IS*26* and close relatives as a sub-group within the IS*6* family.
4. We are surprised Hall feels that “Varani *et al.* also question the existence of TU”, especially since we conclude that “the notion that the basic IS*6* family transposition unit is a non-replicative circular DNA molecule carrying a single IS copy is attractive and would provide a nice parallel to the integron antibiotic resistance gene cassette intermediates”. And also state that “To summarize: it has been clearly demonstrated that circular DNA species carrying a single IS26copy together with flanking “passenger”DNA can be generated efficiently in vivo from a variant plasmid replicon [116] and also that replicons carrying a single IS*26* copy are capable of integrating into a second replicon to form a cointegrate.” We are of the opinion that Harmer et al., have provided considerable evidence for TU (which we inadvertently called “transposable unit” in 2 figures rather than Hall’s “translocatable unit” used in our text). However, again, we express caution in our review because, as yet, there is no firm biochemical data bearing on this interesting and attractive model.

## Conclusion

To understand the true relationship between different members of the IS*6* family and their transposition mechanism(s), it will be essential to extend studies to other family members, particularly those with additional transposase domains (https://tnpedia.fcav.unesp.br/index.php/File:FigIS6.7.png), and to identify physically and biochemically intermediated in their transposition pathways.
